# Recent Advances in Non‐Invasive Digital Nursing Technologies for Chronic Pain Assessment and Management: An Integrative Review

**DOI:** 10.1111/jan.16716

**Published:** 2025-01-10

**Authors:** James Lee, Rebecca Mowat, Julie Blamires, Mandie Foster

**Affiliations:** ^1^ School of Clinical Sciences Auckland University of Technology Auckland New Zealand

**Keywords:** chronic pain, cognitive behavioural therapy, digital technology, internet based intervention, pain management, psychosocial intervention, registered nurses, self management, telemedicine

## Abstract

**Aim:**

This integrative review aims to identify what nurses currently offer through digital technology and their success in managing chronic pain.

**Design:**

An integrative review guided by Whittemore and Knafl was conducted.

**Data Sources:**

Five databases—CINAHL, Medline, PsycINFO, PubMed, and Scopus—were utilised to gather relevant studies from January 2018 to November 2024.

**Review Methods:**

Selected studies were assessed using the Mixed Methods Appraisal Tool and the Joanna Briggs Appraisal Tool. Braun and Clarke's thematic analysis was applied to identify pertinent themes.

**Results:**

Digital nursing technologies such as telehealth and web‐based interventions effectively deliver interventions to assess and manage chronic pain; these technologies can reduce healthcare resource utilisation and increase accessibility. This review highlights that nurses commonly deliver exercise, cognitive‐behavioural therapy, acceptance and commitment therapy and self‐management techniques through digital technology.

**Conclusion:**

This review indicates that web‐based interventions and telemedicine are the primary digital technologies employed by nurses for chronic pain management providing psychosocial interventions, with evidence supporting their effectiveness. Digital and web‐based technology is essential to bridge healthcare access gaps as nurses can provide this successfully with minimal nursing support and cost to the patient.

**Impact:**

Evidence supports nurses in providing psychosocial interventions for the management of chronic pain, particularly web‐based psychosocial interventions. Nurses need to adopt digital technology to improve access to care and patient outcomes and to maintain professional development in an increasingly digital world.

**Patient or Public Contribution:**

No patient or public contribution was used for this study.

## 1 Background

Pain is described as an unpleasant sensory and emotional experience associated with, or resembling that associated with, actual or potential tissue damage (IASP 2021). The aetiology of pain is often complex and not readily explained, making this definition useful for classifying chronic pain. It offers clarity and ease of understanding, making it applicable to conditions like fibromyalgia, irritable bowel syndrome, and back pain, which frequently lack a clear musculoskeletal or neuropathic origin (Treede et al. 2015). The proportions of people experiencing chronic pain are considerable; in a recent large cohort study in the United States, it was estimated that 20.8% (95% CI, 19.6%–21.9%) of the population reported they had chronic pain (Nahin et al. 2023). The personal and societal effects of chronic pain are large, with individuals experiencing associated depression, social breakdown, and stigma, as well as losses related to work productivity and absenteeism (Turk, Wilson, and Cahana 2011).

When it comes to managing chronic pain a multidisciplinary pain approach is considered the gold standard and involves the collaboration of nursing, medicine, physical therapy and mental health professionals, to provide optimal evaluation and management for those suffering from chronic pain (Staudt 2022). This multidisciplinary pain approach is an effective approach because pain is multidimensional, involving social, psychological and physiological factors (Staudt 2022). There is a wealth of foundational research supporting the effectiveness of biopsychosocial approaches and cognitive behavioural models in managing chronic pain patients (Guzmán et al. 2001; Gatchel et al. 2007; Gauthier, Dulong, and Argáez 2019; SIGN 2019; NICE 2021).

While a comprehensive approach involving multiple management elements is preferred for success, individual aspects can be delivered to patients separately. The aim is that changing one aspect will positively impact others, for example, the National Institute for Health and Care Excellence [NICE] (2021) guidelines recommend physical activity to manage chronic primary pain, as it can improve short‐ and long‐term pain and the quality of life of those experiencing chronic pain.

Other effective therapeutic approaches for chronic pain management are Cognitive Behavioural Therapy (CBT) and Acceptance and Commitment Therapy (ACT). CBT is one of the most common forms of psychotherapy treatment for individuals with chronic pain and has the potential to improve the quality of life, pain and activities of daily living (Lim et al. 2018). Cognitive behavioural therapy focuses on finding solutions for current problems and incorporates a wide variety of treatments including relaxation exercises, stress‐reducing and pain‐relieving techniques and problem‐solving strategies, self‐monitoring and relapse prevention. Acceptance and Commitment Therapy is based on mindfulness to empower individuals to accept elements that are out of their control and to engage in behaviours that align with their values (Aytur et al. 2021). It encourages psychological flexibility, which is defined as the ability to recognise and adapt to changing situations, by changing one's attitudes, beliefs and behaviours when there are compromises in social or personal functioning (Aytur et al. 2021). Although ACT is considered a form of CBT, it is distinct from traditional CBT as it focuses on mindfulness, activation and acceptance to facilitate psychological flexibility (Du et al. 2021).

Another important element of chronic pain management is encouraging self‐management. Self‐management refers to the ability of an individual to monitor their health condition and modulate their emotional, cognitive and behavioural responses to achieve a better quality of life (Geraghty et al. 2021). The development of self‐management is achieved through strategies such as self‐reflection, problem‐solving and active goal setting to facilitate behaviour change and enhance self‐efficacy (Devan et al. 2018).

Over the past few years, the development and application of digital technologies in nursing practices have steadily increased (Seibert et al. 2020). Advancements in digital nursing technologies have been driven by technological progress and sociodemographic shifts, such as an ageing population and nursing shortage (Seibert et al. 2020). Digital nursing technologies have the potential to address these challenges by substituting aspects of nursing work, therefore mitigating the rapidly rising costs of care and nursing shortages (Huter et al. 2020). Digital technology such as telehealth has the potential to reduce access barriers such as transportation expenses, treatment availability in remote areas and physical constraints associated with disability (Milosevic et al. 2021). Telehealth can be used by nurses to assess patients remotely, respond to health‐related concerns and provide treatments for chronic pain (Perez et al. 2021). One such example is the utilisation of telehealth to deliver CBT for chronic pain management to increase accessibility (Mayhew et al. 2023).

Digital technologies such as online education, web‐based exercise programmes, web‐based CBT, virtual reality and online self‐management programmes have also been utilised to manage chronic pain (Hussain et al. 2022). Martorella et al. (2017) defined web‐based interventions as programmes that are operated through a website and used to create positive change through the provision of health‐related materials and interactive web‐based components (Hussain et al. 2022). Interventions such as ACT can be provided through a web‐based programme and can either be guided by health professionals or unguided where the patient completes the programme independently (Lin et al. 2017). Virtual reality technology can be used to deliver distraction therapy to reduce pain, by creating a fully (or semi) immersive artificial environment that is delivered through a head‐mounted display (Chaharsoughi, Ahmadifaraz, and Kahangi 2022).

Although evidence‐based guidelines such as the National Institute for Health and Care Excellence (NICE) and Scottish Intercollegiate Guidelines Network (SIGN) on the management of chronic primary pain outline the standards of assessment and management of chronic pain, the role of digital technologies in the delivery of these interventions has not been described (NICE 2021; SIGN 2019). The adoption of a digital technology environment has potential to reduce barriers associated with access to care, thereby improving independence, quality of life and health for patients with chronic pain (Milosevic et al. 2021). Given the pivotal role that nurses play in assessing, managing and evaluating the health progress of consumers across a variety of settings, and the potential that digital technology holds for maximising health and quality of life, nurses must embrace new digital technologies.

As society's reliance on digital technology grows, so does its impact on the nursing profession; therefore, nurses must prepare for the future and advance the profession into the digital age (Booth et al. 2021). By adopting digital technology, nurses can reduce barriers associated with chronic pain by enhancing access to care, resulting in improving independence, quality of life and health (Milosevic et al. 2021). However, the lack of evidence surrounding the effectiveness and use of digital technology in nursing practices impedes the adoption of these technologies (Eriksen and Frandsen 2018). An integrative review of the literature surrounding the role nurses play in managing chronic pain digitally holds potential to enhance evidence‐based practice, improve patient care and facilitate the adoption of digital nursing technologies to manage chronic pain. Therefore, this research asks what digital technology nurses utilise and how successful nurses are in this delivery for managing chronic pain.

The research question is guided by three main aims:

- To examine the role of digital nursing technologies in assessing and managing chronic pain.
- To describe the types of technologies nurses and utilise to manage chronic pain.
- To examine the effectiveness of digital nursing technologies on pain management, patient outcomes and healthcare resource utilisation.

## 2 Methods

Whittemore and Knafl's (2005) integrative review methodology guided this research as it allows findings from diverse research methodologies to inform current clinical and evidence‐based practices. It achieves this by summarising both theoretical and empirical literature to provide a greater understanding of a particular healthcare problem (Whittemore and Knafl 2005). While the combination of studies with diverse methodologies can contribute to the lack of rigour, bias and inaccuracies, the methodology by Whittemore and Knafl (2005) addresses these issues by defining clear systematic and methodological guidelines including problem identification, literature search, data evaluation, data analysis and presentation of findings (Whittemore and Knafl 2005).

Between the 15th of January 2023 and the 12th of November 2024, five databases were searched including CINAHL and MEDLINE through the EBSCOhost platform, PsycINFO through Ovid, PubMed and Scopus. To ensure no records are overlooked, further searches were conducted through Google Scholar (please see Table 1 for search term categories). The database search returned a total of 2016 records from five databases. Subsequently, the records were uploaded to Rayyan, and 450 duplicates were detected through the duplicate detection tool (Ouzzani et al. 2016), resulting in 1556 remaining records.

**TABLE 1:** Search term categories.

Concept	Keywords
Symptom	pain*
	AND
Duration	chronic or persist* or long‐term or long term or prolonged
	AND
Management	assessment or control or evaluation or intervention or
	management or therapy or treatment
	AND
Modality	AI or ‘artificial intelligence’ or computer or digital
	or electronic or internet or online or technology or media
	or tablet or phone or virtual or ‘social media’
	AND
Provider	nurs* or nurse or nursing or nurses
	AND
Design	RCT or randomised control trial or randomized controlled trial

*Wildcard symbol that broadens a search by finding words that start with the same letters.

### 2.1 Inclusion Exclusion Criteria

**FIGURE 1:**
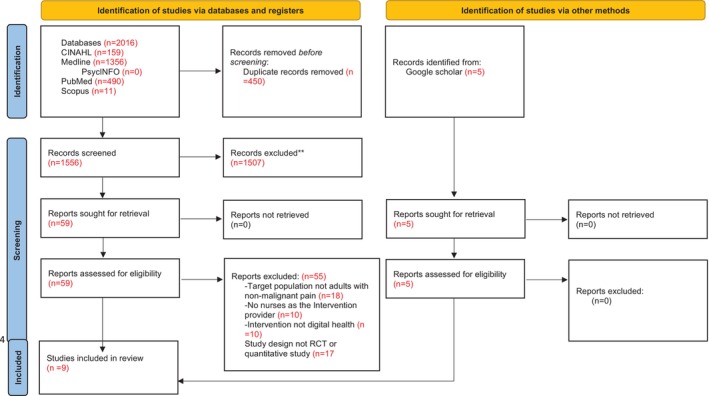
PRISMA 2020 flow diagram for new systematic reviews which included searches of databases, registers and other sources.

The 1556 titles and abstracts were then screened to see if they met the inclusion criteria (see Table 2), resulting in 59 reports. The 59 reports were read through in full and 55 were removed. This resulted in four reports along with five reports found doing a Google search; therefore, nine reports were subjected to full article analysis. Following the database search, the results are presented under the preferred reporting items for systematic reviews and meta‐analyses (PRISMA) flow diagram to enhance transparency and adherence to review methodologies (see Figure 1) (Page et al. 2021).

**TABLE 2:** Inclusion and exclusion criteria.

Criteria	Inclusion	Exclusion
Intervention	Telehealth, computers, smartphones, virtual reality, digital technology	Non‐digital interventions
Target population	Adults > 18 with chronic pain or persistent pain or long‐term pain > 3 months	Age less then < 18 patients without chronic pain. Patients with malignant pain
Intervention provider	Nurses or nurse practitioner	Non‐nurse interventions by doctors, psychologist, allied health
Quantitative studies	Randomised controlled trails	Non‐randomised control trails
Date of publication	Between January 2018 and November 2024	Any study outside these dates
Language	English	All other languages

### 2.2 Data Evaluation

The methodological quality of the randomised control trials included in this review was evaluated with the Mixed Methods Appraisal Tool (MMAT) for randomised controlled trials to exclude low‐quality studies and identify the strengths and limitations of the included studies (Hong et al. 2018). The methodological quality of the systematic review was evaluated using the Joanna Briggs Institute (JBI) critical appraisal checklist to assess the risk of bias in its analysis, conduct and design (Aromataris et al. 2015). To ensure reproducibility and transparency of the integrative review, two authors independently critically appraised the included studies. Additionally, differences in whether an article met the MMAT guideline were resolved through discussion. All articles were found to have met their respective appraisal guideline with 100% compliance.

### 2.3 Data Analysis

As described by Whittemore and Knafl (2005), the data analysis stage of an integrative review is the process in which data from primary sources are coded, categorised, ordered and summarised to provide a comprehensive summary and conclusion that answer the research problem. Additionally, the data analysis methodology included a data display step, which was utilised in this review to populate a table with the aim, author, main results, methodology and intervention of the included studies (see Table 3). To synthesise the findings of this integrative review, the thematic analysis methodology by Braun and Clarke (2006) was utilised. The methodology accomplishes this by analysing the data from the included studies, to identify patterns and themes. This approach was deemed suitable as it enabled the benefits, capabilities and processes associated with digital nursing technologies, as well as the strengths shared by these approaches, to be highlighted.

**TABLE 3:** Summary of selected articles.

Author and year	Title	Aim	Methodology	Sample size and nursing interventions	Main results/ findings
Chen et al. (2022)	The effect of self‐management online modules plus nurse‐led support on pain and quality of life among young adults with irritable bowel syndrome: A randomized controlled trial	To determine the effectiveness of nurse‐led interventions on the quality of life, symptoms and pain levels of patients with irritable bowel syndrome	Randomised controlled trial (Single‐blinded)	Online self‐management education and learning module (*n* = 41) Online self‐management education and learning module with nurse‐led consultations (*n* = 39)	Both groups demonstrated statistically significant (*p* < 0.05) decrease in pain at 12 weeks follow‐up. Nurse‐led intervention group had substantial improvement in quality of life (*p* = 0.040) and reduction in anxiety (*p* = 0.016) when compared to the group without nursing interventions
Diab et al. (2022)	Nurse‐Supported Web‐Based Cognitive Behavioral Therapy for Chronic Musculoskeletal Pain: A Randomized Controlled Trial	To investigate the impact of nurse‐led phone support on pain‐related outcomes in patients receiving web‐based cognitive behavioural therapy	Randomised controlled trial	Web‐based cognitive behavioural therapy with nursing support phone calls (*n* = 30). Web‐based cognitive behavioural therapy alone (*n* = 30)	In both groups, significant improvement in pain intensity and pain interference has been observed. However, there are no significant differences between the control and the interventional group with nursing support
Gannon et al. (2019)	Telehealth therapy effects of nurses and mental health professionals from 2 randomized controlled trials for chronic back pain	Comparing the effectiveness of mental health professionals against primary care nurses delivered telehealth cognitive‐ behavioural therapy and supportive care	Randomised controlled trial. (Single‐blinded)	Telehealth cognitive behavioural therapy and supportive care by a mental health professional (*n* = 66). Telehealth cognitive behavioural therapy and supportive care by a primary care nurse (*n* = 61)	No significant difference in outcomes between the primary care nurse and the mental health professional interventional groups
Gialanella et al. (2020)	Pain, disability and adherence to home exercises in patients with chronic neck pain: Long term effects of phone surveillance. A randomized controlled study	To evaluate the effectiveness of a nurse‐led phone surveillance programme in improving disability, pain management and adherence to neck‐based exercise in neck pain patients. To determine the adherence of the neck‐based exercise at 6 and 12 months after the cessation of phone support	Randomised controlled trial	Patients are allocated to the home‐based surveillance group (*n* = 42) where nurses collect information regarding pain and disability and encourage patients based on that information. In the control group (*n* = 42), patients are advised to continue with the neck exercise with no additional interventions	At six months, neck disability (*p* = 0.012) and pain scores (*p* = 0.013) were lower in the home‐based surveillance intervention group when compared with the control group. Adherence to exercise is 97.6% for the phone intervention group at 6 months and 80.9% for the control group. At 12 months, adherence to exercise is 92.9% in the intervention group and 73.8% for the control group
Gialanella et al. (2017)	Home‐based telemedicine in patients with chronic neck pain	Determining the effectiveness of telemedicine in the reduction of pain in patients with chronic neck pain. This intervention will be directed by physicians and managed by nurses	Prospective randomised controlled trial	The first group of patients was allocated to home‐based telemedicine (*n* = 47). The second group of patients was allocated to home exercise without home‐based telemedicine (*n* = 47)	After 6 months, disability and neck pain declined for both the control and intervention groups (*p* < 0.001). However, neck pain and disability declined significantly more in the telemedicine group when compared to the control group (*p* = 0.001). Adherence at 6x months was at 87.2% for the telemedicine group and 65.9% for the control group
Kroenke et al. (2019)	Automated Self‐management (ASM) vs. ASM‐enhanced collaborative care for chronic pain and mood symptoms: The CAMMPS randomized clinical trial	Comparing the effectiveness of an automated web‐based self‐management intervention (telephone or internet survey) with management by a nurse‐physician team against automated web‐based self‐management intervention only in the treatment of chronic musculoskeletal pain	Randomised controlled trial (Single‐blinded)	The control group (*n* = 147) consists of automated web‐based self‐management intervention only that surveys for symptoms such as pain, depression and anxiety. The intervention group (*n* = 147) consists of automated web‐based self‐management with management by the nurse‐physician team	After 12 months, the z‐score for the control and intervention groups is 0.52 and 0.65 respectively At 12 months, patients in the intervention group are less likely to report worsening of symptoms (19.4%) and reported to be improved (39.5%) when compared to the control group (35.8% worsening, 26.8% improved)
Morcillo‐Muñoz et al. (2022)	Multimodal chronic pain therapy for adults via smartphone: Randomized Controlled Clinical Trial	Determining the effectiveness of a multimodal intervention programme on participants with chronic musculoskeletal pain through the use of internet and mobile devices	Randomised controlled trial	The intervention group (*n* = 98) will utilise the smartphone NO+Dolor (translated from Spanish: no pain) app for 6 weeks, with participants receiving 3 activities (psychosocial therapy programme) each week. The control group (*n* = 96) only has access to the ‘find out more’ section of the app, which contains video information on self‐help approaches	There are significant positive differences in the intervention group when compared to the control group in quality of life (*p* = 0.002), acceptance (*p* = 0.001), rumination (*p* < 0.001), catastrophising (*p* < 0.001) and helplessness (*p* = 0.002)
Rutledge et al. (2018)	Randomized controlled trial of nurse‐delivered cognitive‐behavioral therapy versus supportive psychotherapy telehealth interventions for chronic back pain	To determine the effectiveness of nurse‐delivered telehealth cognitive behavioural therapy or supportive psychotherapy	Randomised controlled trial	Participants allocated to the cognitive behavioural therapy telehealth group (*n* = 33) was conducted by nurses focusing on self‐management and behavioural change (lessons and exercises)	Both cognitive behavioural therapy and supportive psychotherapy groups demonstrated statistically significant improvement in pain, severity, back pain disability and patient‐rated improvements, with moderate effect size. Comparison between the groups demonstrated no significant differences in any measured aspects
Skolasky et al. (2024)	Nurse‐led web‐based self‐management program to improve patient activation and health outcomes in patients with chronic low back pain: an acceptability and feasibility pilot study	To test an intervention that incorporates evidence‐based strategies to improve patient activation in an effort to tailor self‐management strategies to people living with cLBP and to determine the potential for a larger clinical trial in this population	Randomised controlled trial (Single‐masked)	Control (*n* = 12), self‐management programme (*n* = 4) or self‐management programme (SMP) + health behavioural change counselling (HBCC) (*n* = 18)	Participants in the SMP + HBCC groups had at least medium effect size improvements in Patient Activation Measures and small‐to‐medium effect size improvements in Oswestry Disability Index scores and physical function and large effect size improvement in social roles at 12 weeks. Improvements persisted in the SMP + HBCC group at 26 weeks

The first phase of thematic analysis involves familiarisation of the data, which was achieved by actively, analytically and critically reading and rereading the included articles until a comprehensive understanding of the data content was achieved (Braun and Clarke 2006). This process was supported by the table generated in the data display step, which provided contextual information and served as a template for generating initial codes in the second phase of the analysis (Braun and Clarke 2006). This process was conducted electronically by extracting the data to Microsoft Word and coding extracts of data from the studies that were relevant to the integrative review question. These codes were reviewed for similarities and overlaps to generate themes and subthemes in the third phase of the analysis (Braun and Clarke 2006). In the fourth phase, the themes were reviewed for quality and quantity in relation to the review question and data sets (Braun and Clarke 2006). Boundaries of the themes were defined, and their coherence was assessed to ensure that the data was not excessively diverse. In the fifth phase, the themes were named, defined and carefully assessed for their purpose, scope and focus (Braun and Clarke 2006). Lastly, the themes were analysed and in a logical and compelling narrative and presented as the findings of this integrative review (Braun and Clarke 2006).

### 2.4 Findings

Overall, nine studies were included in this integrative review, consisting of nine randomised control trials. Of the nine randomised control trials, six were based in the United States (Chen et al. 2022; Diab et al. 2022; Gannon et al. 2019; Kroenke et al. 2019; Rutledge et al. 2018; Skolasky et al. 2024), two in Italy (Gialanella et al. 2020, 2017), and one in Spain (Morcillo‐Muñoz et al. 2022).

The demographic characteristics from the RCT interventional group consisted of 82.1% females with a mean age of 21.2 (Chen et al. 2022), 86.7% females with a mean age of 52.3 (Diab et al. 2022), 89.5% females with a mean age of 54.6 (Gialanella et al. 2020), 88.1% females with a mean age of 56.0 (Gialanella et al. 2017), 80.0% females with a mean age of 51.2 (Morcillo‐Muñoz et al. 2022), 88.2% males with a mean age of 62.4 (Gannon et al. 2019), 87.4% males with a mean age of 57.4 (Kroenke et al. 2019), 87.0% males with a mean age of 62.5 (Rutledge et al. 2018), and 56% females with a mean age of 45 (Skolasky et al. 2024).

Analysis of the nine included studies resulted in four key themes that explored and elucidated how digital nursing technologies are used in the management of chronic pain. The results revealed what current digital technology is available to nurses, what chronic pain management techniques nurses can currently successfully deliver through digital technology to enhance accessibility, the role nurses play in enhancing self‐management alongside digital technology and the effectiveness of nurse delivered digital technology.

### 2.5 Current Digital Technology Available to Nurses

This theme describes the potential use of digital technology by nurses and the diverse range of digital technology available for the assessment and management of chronic pain. All studies investigated the use of digital technology by nurses in the management of chronic pain (Chen et al. 2022; Diab et al. 2022; Gannon et al. 2019; Gialanella et al. 2020; Gialanella et al. 2017; Kroenke et al. 2019; Morcillo‐Muñoz et al. 2022; Rutledge et al. 2018; Skolasky et al. 2024).

In three of the reviewed articles, nurses utilised phone‐based telehealth to monitor patients with chronic neck pain, musculoskeletal pain and lower back pain (Gialanella et al. 2017; Gialanella et al. 2020; Skolasky et al. 2024). Monitoring consisted of scheduled fortnightly phone calls by a nurse over 6 months, who collected information on disability, pain, analgesic use and adherence to rehabilitative exercises (Gialanella et al. 2017, 2020). Based on the received information, the nurse provided advice to alleviate the exacerbation of symptoms and persistent pain, and patients were also encouraged to perform rehabilitative exercises. Furthermore, a rehabilitation specialist was consulted in cases where a second opinion was required, who provided indications for pharmacological interventions, specific exercises and postural education (Gialanella et al. 2017, 2020). In one arm of Skolasky et al.'s (2024) study nurses delivered a web‐based 6‐week self‐management programme followed up with telephone‐based health behavioural change counselling to increase patient activation and self‐management behaviour, reduce pain and disability and improve quality of life. The telephone sessions were delivered once before the programme commenced and two times during the self‐management programme.

In addition to monitoring, telehealth was utilised in two studies to deliver cognitive‐behavioural therapy to manage patients with chronic low back pain (Gannon et al. 2019; Rutledge et al. 2018). Eight weekly telehealth CBT sessions were delivered by a nurse, and each session was accompanied by written instructions on self‐monitoring exercises, homework exercises and educational information. The CBT sessions explored topics including self‐management, communication, pain education and health behaviours, which improved pain intensity and back disability (Gannon et al. 2019; Rutledge et al. 2018).

Kroenke et al. (2019) explored web‐based programmes utilised by nurses to monitor patient symptoms and provide interventions, where patients being monitored were required to complete internet‐based symptom surveys on pain, depression, anxiety, fatigue, impairment and sleep. Once the survey was submitted, data from the survey prompted the use of mood and pain self‐management modules on problem‐solving, depression, anger management and sleep (Kroenke et al. 2019). In addition, a comprehensive management strategy was employed, where patients were supported by a nurse‐physician team (Kroenke et al. 2019). In this management strategy, nurses are alerted by e‐mail based on the information that is submitted in the symptom surveys. Items that generate e‐mail alerts include non‐adherence to medication, missed surveys and requests for treatment change. Based on the alerts and the tabulated surveys, the nurse‐physician team offered patients further advice, and the option of analgesia, psychotropic medications and referral to psychologists (Kroenke et al. 2019). Web‐based monitoring shares many similarities with telehealth monitoring. Firstly, both modalities were utilised to gather symptom information from the patient. Secondly, the gathered information was used to inform a response. Lastly, the nurse consulted a medical specialist for further advice.

One study demonstrated that web‐based CBT can effectively reduce pain intensity and pain interference associated with chronic musculoskeletal pain (Diab et al. 2022). In this study, patients had access to the painTRAINER programme, containing eight learning modules on topics such as activity/rest cycles, problem‐solving and relapse prevention. This intervention included phone‐based support from nurses, who provided motivational support through motivational interviewing to increase engagement (Diab et al. 2022). Similarly, web‐based programmes were utilised by nurses to deliver self‐management modules and online education in two studies (Chen et al. 2022; Skolasky et al. 2024). These online modules contain videos on topics relating to pain neurophysiology, self‐management strategies and advice regarding physical activity (Chen et al. 2022). Further Skolasky's study provided modules over six weeks on self‐management based on arthritis and chronic disease evidence‐based self‐management programmes and focused on connection, communication with others, healthy eating and weight management, medication management and planning for the future (Skolasky et al. 2024). Additionally, nurses utilised phone‐based telehealth to support patients in creating their self‐management goals and overcoming barriers associated with their goals (Chen et al. 2022; Skolasky et al. 2024). This demonstrates that predominantly telehealth and web‐based programmes were utilised by nurses to deliver interventions such as CBT and self‐management education to manage chronic pain.

### 2.6 Chronic Pain Management Techniques Nurses Can Successfully Deliver Through Digital Technology to Enhance Accessibility

Seven studies investigated the role of nurses in enhancing the accessibility of chronic pain treatments by delivering chronic pain education via digital technology that may not be available through other modes of contact (Chen et al. 2022; Diab et al. 2022; Gannon et al. 2019; Gialanella et al. 2020; Kroenke et al. 2019; Morcillo‐Muñoz et al. 2022; Skolasky et al. 2024). Nurses can increase access to psychosocial therapies such as CBT and ACT, which is important as these therapies are effective in managing chronic pain (Diab et al. 2022; Morcillo‐Muñoz et al. 2022).

Considering the practical benefit of utilising existing nursing staff in primary care clinics, Gannon et al. (2019) investigated if nurses can effectively deliver telehealth CBT to manage chronic pain. This study compared telehealth CBT delivered by trained nurses to delivery by doctoral‐level mental health professionals (Gannon et al. 2019). The results from this study demonstrated no significant differences in the Roland‐Morris Disability Questionnaire (RMDQ), Numeric Pain Rating Scale (NRS) or Pain Catastrophizing Scale between the two groups of professionals (*p* > 0.05) (Gannon et al. 2019). This demonstrated that nurses can successfully enhance accessibility to chronic pain treatments by delivering web‐based CBT.

Similarly, when nurses were delivered unguided web‐based self‐management programmes along with three sessions of nurse‐led one‐to‐one consultation plus self‐management online education and learning modules to manage irritable bowel syndrome pain, there was a significant reduction in patients reported pain intensity (*p* < 0.05) and pain interference (*p* < 0.05) (Chen et al. 2022). In addition, this guided nurse consultation demonstrated statistically significant improvements in quality of life (*p* = 0.040) and reduction in anxiety (*p* = 0.016) (Chen et al. 2022).

As shown above, evidence‐based treatments such as web‐based CBT can be used to assist people with chronic pain; however, unlike telehealth CBT, web‐based CBT can be delivered unguided (Diab et al. 2022). Diab et al. (2022) compared the effectiveness of unguided web‐based CBT with nurse‐guided web‐based CBT and demonstrated no differences in pain intensity or pain interference between the groups of patients with chronic pain (Diab et al. 2022). Although this study did not specifically demonstrate the role of nurses in delivery per se, it did demonstrate that web‐based CBT can be effective, therefore reducing healthcare resource utilisation.

As described previously, telehealth can be used by nurses to monitor patients with chronic pain and provide advice to alleviate symptoms of exacerbation and persistent pain (Gialanella et al. 2020; Skolasky et al. 2024). Telehealth monitoring enables nurses to improve access to chronic pain treatments, enabling remote access for patients with mobility disabilities and travel constraints. The use of web‐based automated monitoring by nurses has the potential to further reduce healthcare resource utilisation and enhance accessibility (Kroenke et al. 2019). Kroenke's study described the use of web‐based automated monitoring, where patients submit an online survey on their symptoms, adherence to exercise and medication use. Based on the submitted information (or absence of submission), email alerts were generated and sent to nurses, who then contacted the patient to address these concerns. Compared to telehealth monitoring, the use of web‐based automated monitoring reduces healthcare resource utilisation, as nurses are not required to conduct weekly telehealth symptom surveys.

Skolasky et al.'s (2024) randomised controlled trail investigated the delivery of self‐management topics over 6 weeks which included an overview of self‐management specific for chronic low back pain, mind‐body connection, communication with others, healthy eating and weight management, medication management and planning for the future. In one arm of Skolasky's study participants received the above 6 weeks of education along with three telephone calls which consisted of information based on a health behaviour change plan along with two booster conversions (Skolasky's et al. 2024).

### 2.7 Nurses Enhancing Self‐Management Alongside Digital Technology

This review has demonstrated the important role that nurses play delivering chronic pain techniques using digital technology. It also highlights how the use of digital technology by nurses can also be utilised to enhance self‐management strategies. In eight of the included articles, nurses utilised digital interventions to deliver self‐management education and practical skills to manage chronic pain (Diab et al. 2022; Chen et al. 2022; Gannon et al. 2019; Morcillo‐Muñoz et al. 2022; Rutledge et al. 2018; Gialanella et al. 2017; Gialanella et al. 2020; Skolasky et al. 2024). Self‐management is defined as a process where individuals are empowered as the central decision‐maker, where they utilise their knowledge and beliefs, social facilitation and self‐regulation skills to achieve health‐related outcomes (Chen et al. 2022).

In cognitive behavioural therapy, behaviour change and self‐management are crucial in modifying negative thinking patterns and maladaptive behaviours, as these processes can exacerbate functional impairment and pain intensity (Diab et al. 2022; Gannon et al. 2019). Self‐management plays a major role in CBT, two studies utilised telehealth and web‐based CBT to deliver self‐management to reduce pain intensity and pain interference, and in the Roland‐Morris Disability Questionnaire (RMDQ) (Diab et al. 2022; Gannon et al. 2019). In web‐based CBT, self‐management modules on activity/rest cycles, coping thoughts, relapse prevention and problem‐solving were delivered as part of the web‐based CBT intervention (Diab et al. 2022). Furthermore, nurses delivered telehealth CBT self‐management sessions on goal setting, stress management, changing self‐talk and sleep hygiene (Gannon et al. 2019). This demonstrates the importance of self‐management in CBT interventions for chronic pain management.

Moricillo‐Munoz et al. utilised a web‐based smartphone or mobile device app to evaluate the effectiveness of a multimodal intervention programme to manage patients with chronic pain (Morcillo‐Muñoz et al. 2022). The study used web‐based Acceptance and Commitment Therapy (ACT) which incorporates interactive mindfulness activities and exercises to raise awareness of one's values, helping patients to observe and recognise pain‐related emotions and thoughts while promoting pain acceptance. Additionally, the web‐based application includes a dedicated exercise section with walking, exercise and stretching activities aimed at enhancing physical and emotional well‐being (Morcillo‐Muñoz et al. 2022). Web‐based self‐management sessions delivered over 6 weeks reinforced with phone calls from nurses were used to evaluate patient activation and pain‐related disability (Skolasky et al. 2024).

Through nursing‐based phone surveillance, three studies demonstrated that monitoring can enhance self‐management to reduce neck pain, disability and lower back pain (Gialanella et al. 2017; Gialanella et al. 2020; Skolasky et al. 2024). These studies facilitate self‐management through regular monitoring, allowing nurses to encourage patients to exercise regularly and provide feedback and support, which in turn increases adherence, motivation and self‐efficacy, even after the cessation of the intervention (Gialanella et al. 2020; Skolasky et al. 2024). This theme demonstrated the importance of self‐management in chronic pain interventions, by highlighting the ways nurses utilised digital technology to provide education and practical skills to manage chronic pain.

### 2.8 The Effectiveness of Nurse Delivered Digital Technology

The effectiveness of digital nursing technologies for the assessment and management of chronic pain was evaluated in all of the studies used for this review. Success varied and depended on the types of digital interventions delivered. Three studies investigated the benefits of telehealth surveillance in the management of chronic pain (Gialanella et al. 2017; Gialanella et al. 2020; Skolasky et al. 2024). In two studies, telehealth surveillance consisted of fortnightly scheduled phone calls, where a nurse collected information on pain, rehabilitative exercise adherence, symptom exacerbation and medication use (Gialanella et al. 2017, 2020). Also, the nurse encouraged the patients to perform rehabilitative exercises and provided clinical feedback based on the collected information. Results from Gialanella et al. (2017) and Gialanella et al. (2020) demonstrated a statistically significant reduction in neck disability index (NDI) (*p =* 0.012) (*p =* 0.001) and pain visual analogue scale (VAS) (*p =* 0.013) (*p =* 0.001), respectively, compared to controls without nursing support.

Telehealth was used to enhance health behaviour change reinforcing and supporting a 6‐week self‐management programme (Skolasky et al. 2024). Participant results showed those receiving both the 6‐week self‐management programme and the phone health behaviour change input had approximately a 4‐point improvement in patient activation at the 12‐ and 26‐week assessments; these improvements were of medium‐to‐large effect size compared to those in the control group and of medium effect size compared to those in the self‐management programme only (Skolasky et al. 2024). Those in both the self‐management programme and the self‐management programme with health behavioural change telephone follow‐up also reported improvement in physical function and social roles and reduction in pain‐related disability and pain interference at the 12‐ and 26‐week assessments. Effects were larger in the group that received the telephone behavioural change counselling (Skolasky et al. 2024).

In another study, a web‐based self‐management intervention was delivered to manage chronic musculoskeletal pain (Kroenke et al. 2019). Patients were monitored by nurses through a patient‐submitted web‐based online survey, and advice was given by nurses based on the submitted information. The study investigated the effects of the web‐based self‐management intervention with and without the addition of multidisciplinary nursing support (Kroenke et al. 2019). Results from this study demonstrated a significant reduction in pain‐anxiety‐depression (PAD) score (*p =* 0.003) when nursing support was added to the web‐based self‐management intervention. Chen et al. (2022) investigated the addition of guided nursing support in the web‐based self‐management module intervention, demonstrating significant improvements in quality of life (*p =* 0.040) and reduction of anxiety (*p =* 0.016). These studies demonstrated that guided nursing support and monitoring are valuable additions to web‐based self‐management interventions, due to their potential to reduce PAD and anxiety and improve quality of life. However, Diab et al. (2022) investigated the addition of nurse‐delivered telehealth motivational interviewing to support web‐based CBT, which demonstrated no significant difference in Brief Pain Inventory (BPI) measures for pain interference (*p* > 0.05) and pain intensity (*p* > 0.05), when compared to controls without nursing support. This demonstrates that nurses may not be needed to deliver a successful web‐based intervention.

Three studies investigated the long‐term effects of digital nursing technology to evaluate their sustained impacts, which is an important factor when considering interventional frequency and effectiveness (Diab et al. 2022; Gialanella et al. 2020; Morcillo‐Muñoz et al. 2022). Diab et al. (2022) demonstrated a statistically significant reduction in the BPI pain interference score (*M* = −1.3, 95% CI = −2.0, −0.7, *p* < 0.05) and pain intensity (*M* = −1.2, CI = −1.7, −0.6, *p* < 0.05) 8 weeks after the cessation of web‐based CBT with nursing support, with similar results in pain interference score (*M* = −1.7, CI = −2.3, −1.0, *p* < 0.05) and pain intensity (*M* = −1.3, CI = −1.8, −0.8, *p* < 0.05) without nursing support. Another study by Morcillo‐Muñoz et al. (2022) demonstrated a statistically significant reduction in catastrophising (*p <* 0.05), helplessness (*p <* 0.05) and rumination (*p <* 0.05) 3 months after the discontinuation of web‐based psychosocial therapy when compared to the control group without psychosocial intervention. These studies indicate that the treatment effects resulting from web‐based psychosocial therapies can last from 2 to 3 months, reducing both psychosocial and physical effects stemming from chronic pain. In addition to these findings, telehealth monitoring has been shown to significantly improve the neck disability index (*p =* 0.026) and adherence to rehabilitative exercises (χ^2^ = 5.485, *p* = 0.019) for 6 months after the cessation of monitoring, with 92.9% of patients performing two to seven rehabilitation sessions each week from a baseline of 97.6% (Gialanella et al. 2020). In comparison, the control group exhibited an adherence rate of 73.8% after 6 months, compared to a baseline of 92.9%. This demonstrates that nursing support through monitoring has a significant impact on adherence rate.

The effectiveness of digital technologies could be affected by the type of psychosocial therapy delivered and the type of healthcare personnel delivering the intervention. One study investigated nurse‐delivered CBT and supportive psychotherapy as a control for nonspecific factors such as treatment frequency and duration, which demonstrated no significant differences in the Roland‐Morris Disability Questionnaire (RMDQ) score (2.0‐point decrease for CBT and SC) (*F* = 0.05, 95% CI = −3.3, 2.6, *p* = 0.84) and the numeric rating scale (NRS) for pain (0.9‐point decrease for CBT and 1.2‐decrease for SC) (*F* = 0.16, CI = −1.2, 0.83, *p* = 0.70) between the interventions with comparable effect sizes (Rutledge et al. 2018). A follow‐up study concluded that there were no statistically significant differences in RMDQ (*p* > 0.05) or NRS (*p* > 0.05) when CBT or SC was delivered by either a primary care nurse or mental health professionals (Gannon et al. 2019).

## 3 Discussion

As society increases its reliance on digital technologies, so does its dependence on virtual models of care (Booth et al. 2021). The utilisation of telehealth to deliver CBT allows nurses to improve treatment access for those living in rural areas, areas without specialist or healthcare services, and to low socio‐economic communities (Bashir and Bastola 2018). Therefore, nurses must prepare for the future by keeping up with the rapid advancements in technology, to enhance patient care and support nursing practices (Booth et al. 2021). To facilitate the nursing profession into the digital age, this review explored what is the most up‐to‐date digital technology used to deliver chronic pain management by nurses, what types of chronic pain management methods are currently used, and finally this review explored the success of such techniques in the management of chronic pain.

### 3.1 Current Digital Technology Available to Nurses

The findings of this review revealed that nurses primarily deliver chronic pain management through phone‐based telehealth and web‐based interventions (Diab et al. 2022; Gialanella et al. 2020; Skolasky et al. 2024). Each technology has the potential to deliver a wide range of interventions such as CBT, ACT, patient monitoring and self‐management education (Morcillo‐Muñoz et al. 2022). This review found that nurses were able to utilise telehealth to monitor and advise patients with chronic neck and low‐back pain (Gannon et al. 2019; Gialanella et al. 2020; Skolasky et al. 2024). And nurses can utilise web‐based interventions to deliver CBT and self‐management modules (Chen et al. 2022; Kroenke et al. 2019; Skolasky et al. 2024). Nurses were also able to utilise web‐based programmes to facilitate patient monitoring and provide clinical advice (Kroenke et al. 2019; Morcillo‐Muñoz et al. 2022; Skolasky et al. 2024).

In New Zealand, the ‘Just A Thought’ website provides web‐based CBT for mental health disorders such as generalised anxiety and depression (Mahoney et al. 2021). No CBT resources directly linked to chronic pain are available; however, self‐care resources offered by Healthify He Puna Waiora (2024) provide health information. Similarly, the New Zealand Pain Society (2024) have a resource called navigating pain which provides tools such as encouraging physical activity, sleep, mindfulness and relaxation.

An Australian web‐based CBT programme ‘THIS WAY UP’ currently offers free web‐based CBT for chronic pain, depression and anxiety, for those under the supervision of a healthcare professional such as nurses and general practitioners (Mahoney et al. 2021). The programme can be used to deliver online psychological education, through a series of illustrated comic‐style lessons that follow a story of a fictional character experiencing mental health challenges. Nurses can track their patients' progress through the website, and the interventions can either be guided or unguided. This demonstrates that web‐based CBT is readily available for nurses to promote the health and well‐being of those suffering from chronic pain.

### 3.2 Chronic Pain Management Techniques Nurses Can Successfully Deliver Through Digital Technology to Enhance Accessibility

Findings from this review indicate that telehealth CBT can be effectively delivered by nurses to manage chronic pain, demonstrating comparable effectiveness with other mental health professionals (Gannon et al. 2019). This is consistent with findings from research, which support the utilisation of nurses to deliver web‐based CBT, leading to improved pain‐related outcomes (Ehde, Dillworth, and Turner 2014). Due to the high prevalence of chronic pain and escalating demand for mental health services, practice nurses are appropriately placed to manage patients with chronic pain.

In addition, the findings from this review indicated that web‐based CBT and self‐management interventions can be utilised to increase access and reduce healthcare resources (Chen et al. 2022; Kroenke et al. 2019; Morcillo‐Muñoz et al. 2022). These web‐based applications facilitate automated monitoring, allowing nurses to track and monitor the clinical status of their patients (Kroenke et al. 2019; Morcillo‐Muñoz et al. 2022). As demonstrated in the findings of this review, the main benefit of web‐based CBT and self‐management stems from its ability to deliver interventions effectively with little to no nursing support (Chen et al. 2022; Diab et al. 2022; Kroenke et al. 2019). Studies have shown that web‐based CBT has the potential to increase patients' access to CBT, by overcoming barriers such as mobility, location, time constraints and the stigma of therapy (Guliani et al. 2022). Web‐based CBT can be scaled up and rapidly disseminated to patients experiencing chronic pain, with little to no cost to patients (Morgan et al. 2017). As mentioned above the web‐based CBT programme ‘THIS WAY UP’ is a free web‐based CBT programme, funded by the Australian Department of Health, and nurses worldwide can prescribe these courses to their patients and monitor their progress through the web interface (Mahoney et al. 2021). However, access to web‐based CBT may be challenging for those with limited technological proficiency, disabilities and language barriers (Newby et al. 2021).

### 3.3 Nurses Enhancing Self‐Management Along Side Digital Technology

Results from this review demonstrated that self‐management plays a significant role in chronic pain interventions as shown in five studies (Chen et al. 2022; Diab et al. 2022; Morcillo‐Muñoz et al. 2022; Gannon et al. 2019; Gialanella et al. 2020; Skolasky et al. 2024). In these studies, nurses utilised digital technology to provide learning modules, interactive psychosocial mindfulness activities, self‐management programmes, monitoring and exercise activities to enhance self‐management. Due to the persistent nature of chronic pain, it is important to promote the self‐management aspect of chronic pain as it improves the quality of life, health status and management of pain (Hestmann, Bratås, and Grønning 2023).

Chen et al. (2022) described self‐management as a process where individuals are empowered as the central decision‐maker, where they utilise their knowledge and beliefs, social facilitation, and self‐regulation skills to achieve health‐related outcomes. Based on this definition, it is clear that social facilitation and self‐regulation skills are critical components of self‐management. In terms of social facilitation, it is defined as the improved performance accuracy and the level of exertion in the presence of others (Chib, Adachi, and O'Doherty 2018). Social facilitation is one of many factors that contribute to the enhanced efficacy of guided digital interventions over unguided digital interventions in chronic pain management (Lin et al. 2017). Therefore, nurses should promote social facilitation by guiding patients through self‐management modules, activities, and programmes to enhance self‐management and achieve better health outcomes. Additionally, nurses can incorporate effective communication and empathy during the guided sessions to promote a collaborative relationship (Kerns et al. 2022).

### 3.4 The Effectiveness of Nurse Delivered Digital Technology

The findings of this integrative review demonstrated that nursing‐based telehealth surveillance can significantly improve pain scores, disability and adherence to rehabilitation exercises (Gialanella et al. 2017; Gialanella et al. 2020; Skolasky et al. 2024). The results from this review indicated that web‐based self‐management interventions are effective at reducing pain‐related outcomes (Chen et al. 2022; Kroenke et al. 2019; Skolasky et al. 2024). The addition of telehealth nursing support to web‐based self‐management interventions significantly improved PAD score, anxiety and quality of life (Chen et al. 2022; Kroenke et al. 2019), and this was also seen with Skolasky et al. (2024), mentioned in this study, who found improved patient activation, social roles and physical function along with a reduction in pain‐related disability and pain interference at 12‐ and 26‐week assessments when self‐management skills were delivered in combination with follow‐up phone calls. This observation is concordant with the findings from Chew et al. (2023), which concluded that web interventions with guided support (regular interaction with healthcare staff) are superior to unguided web interventions. However, one study from this review demonstrated that the addition of motivational interviewing nursing support to web‐based CBT did not improve pain interference (*p* > 0.05) or pain intensity (*p* > 0.05) (Diab et al. 2022). There could be a few explanations for this observation. Firstly, the study did not assess the treatment fidelity of the MI‐based nursing support, which means that there is a possibility that motivational interviewing was not delivered by nurses (Diab et al. 2022). Secondly, the failure to meet statistical significance could be explained by the small sample size of the study, due to the relationship between the *p* value (statistical significance), sample size and the magnitude of association between the variables (Kostis and Dobrzynski 2020). Finally, prior knowledge of the treatment group assignment by patients may have contributed to bias, potentially underestimating the treatment effects of MI‐based nursing support (Diab et al. 2022).

The results from this review demonstrated that nurses can effectively provide telehealth CBT and supportive psychotherapy to manage chronic pain (Rutledge et al. 2018). However, there were no significant differences in Roland‐Morris Disability Questionnaire score or Numeric Pain Rating Scale between the provided interventions (Rutledge et al. 2018). It has been hypothesised that nonspecific factors such as treatment time, treatment frequency and therapeutic relationship found in both forms of psychotherapy contribute to their effectiveness, resulting in similar outcomes (Rutledge et al. 2018). This phenomenon is described by the Dodo Bird Verdict, which states that all forms of psychotherapy are comparable in effects with no significant differences between one another (Cuijpers, Reijnders, and Huibers 2019). Accordingly, nurses can utilise the nonspecific factors to enhance nursing practices and the effects of psychotherapy, by promoting a stronger nurse–patient therapeutic alliance and interventional frequency to improve patient outcomes (Zilcha‐Mano et al. 2019). The use of digital technologies can improve nurse–patient alliance and increase interventional frequency by reducing geographical limitations between nurses and patients (Rejula et al. 2021).

### 3.5 Strengths

- This review described and evaluated digital technologies that have been utilised by nurses to facilitate evidence‐based practice and data were collected between 2018 and 2024, to demonstrate current evidence.
- The pragmatic nature of this review informs nurses of the practical benefits and practicalities of digital technology in chronic pain assessment and management.
- This utilisation of the integrative review methodology enhances the rigour of the study as the methodologies are clearly outlined, and the included studies were evaluated by two people for methodological quality.
- As the integrative review methodology was utilised in this study, the results and synthesis of this review apply to practice.

### 3.6 Limitations

- Findings from this review are only applicable to adults due to the patient characteristics of the included studies and the aim of the integrative review.
- As this review focuses on digital technologies that were utilised by nurses, there could be digital technologies that are more effective and cost‐effective than the ones described in this review that were not utilised by nurses.
- Although a systematic approach was used to search for relevant studies, there is a possibility that some studies were missed.
- Although this study chose an integrative review that could include both qualitative and quantitative studies, we only used quantitative studies so missed exploring the lived experiences of practitioners and their use of digital technologies.

## 4 Conclusion

This review demonstrates that web‐based interventions and telemedicine are currently the most utilised digital technology used by nurses for chronic pain management, and there is supporting evidence of their success. Digital and web‐based technology and telehealth are important to bridge gaps in healthcare access as it can be provided successfully by nurses with minimal nursing support and cost to the patient. This review shows that exercise, cognitive behavioural therapy, acceptance and commitment therapy and self‐management modalities are most often delivered via digital technology to help with chronic pain management. These techniques combined with nurses who monitor and support the patient with chronic pain have the potential to increase the efficacy of digital interventions.

## Conflicts of Interest

The authors declare no conflicts of interest.

## Peer Review

The peer review history for this article is available at https://www.webofscience.com/api/gateway/wos/peer‐review/10.1111/jan.16716.

## Supporting information


Appendix S1.

## Data Availability

The authors have nothing to report.

## References

[jan16716-bib-0001] Aromataris, E. , R. Fernandez , C. Godfrey , C. Holly , H. Khalil , and P. Tungpunkom . 2015. “Summarizing Systematic Reviews.” International Journal of Evidence‐Based Healthcare 13, no. 3: 132–140. 10.1097/xeb.0000000000000055. 26360830

[jan16716-bib-0002] Aytur, S. , K. Ray , S. Meier , et al. 2021. “Neural Mechanisms of Acceptance and Commitment Therapy for Chronic Pain: A Network‐Based Fmri Approach.” Frontiers in Human Neuroscience 15: 587018. 10.3389/fnhum.2021.587018. 33613207 PMC7892587

[jan16716-bib-0003] Bashir, A. , and D. Bastola . 2018. “Perspectives of Nurses Toward Telehealth Efficacy and Quality of Health Care: Pilot Study.” JMIR Medical Informatics 6, no. 2: 9080. 10.2196/medinform.9080. PMC5993972 29802089

[jan16716-bib-0004] Booth, R. G. , G. Strudwick , S. McBride , S. O'Connor , and A. L. Solano López . 2021. “How the Nursing Profession Should Adapt for a Digital Future.” BMJ 373: n1190. 10.1136/bmj.n1190.

[jan16716-bib-0005] Braun, V. , and V. Clarke . 2006. “Using Thematic Analysis in Psychology.” Qualitative Research in Psychology 3, no. 2: 77–101. 10.1191/1478088706qp063oa.

[jan16716-bib-0006] Chaharsoughi, N. , M. Ahmadifaraz , and L. Kahangi . 2022. “The Impact of Digital Technologies in Nursing Care and Their Application: A Narrative Review.” Journal of Multidisciplinary Care 11: 149–156. 10.34172/jmdc.2022.1127.

[jan16716-bib-0007] Chen, J. , Y. Zhang , Z. Barandouzi , et al. 2022. “The Effect of Self‐Management Online Modules Plus Nurse‐Led Support on Pain and Quality of Life Among Young Adults With Irritable Bowel Syndrome: A Randomized Controlled Trial.” International Journal of Nursing Studies 132: 104278. 10.1016/j.ijnurstu.2022.104278. 35640500 PMC10588769

[jan16716-bib-0008] Chew, M. , C. Chan , S. Kobayashi , H. Cheng , T. Wong , and L. Nicholson . 2023. “Online Pain Management Programs for Chronic, Widespread Musculoskeletal Conditions: A Systematic Review With Meta‐Analysis.” Pain Practice 23: 664–683. 10.1111/papr.13227. 37051894

[jan16716-bib-0009] Chib, V. , R. Adachi , and J. O'Doherty . 2018. “Neural Substrates of Social Facilitation Effects on Incentive‐Based Performance.” Social Cognitive and Affective Neuroscience 13, no. 4: 391–403. 10.1093/scan/nsy024. 29648653 PMC5928408

[jan16716-bib-0010] Cuijpers, P. , M. Reijnders , and M. Huibers . 2019. “The Role of Common Factors in Psychotherapy Outcomes.” Annual Review of Clinical Psychology 15, no. 1: 207–231. 10.1146/annurev-clinpsy-050718-095424. 30550721

[jan16716-bib-0014] Devan, H. , L. Hale , D. Hempel , B. Saipe , and M. A. Perry . 2018. “What Works and Does Not Work in a Self‐Management Intervention for People With Chronic Pain? Qualitative Systematic Review and Meta‐Synthesis.” Physical Therapy 98, no. 5: 381–397. 29669089 10.1093/ptj/pzy029

[jan16716-bib-0015] Diab, R. , R. Bomar , J. Slaven , S. Kaplan , and D. Ang . 2022. “Nurse‐Supported Web‐Based Cognitive Behavioral Therapy for Chronic Musculoskeletal Pain: A Randomized Controlled Trial.” Pain Physician 25, no. 7: 959–968. 36288581

[jan16716-bib-0016] Du, S. , J. Dong , S. Jin , H. Zhang , and Y. Zhang . 2021. “Acceptance and Commitment Therapy for Chronic Pain on Functioning: A Systematic Review of Randomized Controlled Trials.” Neuroscience & Biobehavioral Reviews 131: 59–76. 10.1016/j.neubiorev.2021.09.022. 34536462

[jan16716-bib-0017] Ehde, D. , T. Dillworth , and J. Turner . 2014. “Cognitive‐Behavioral Therapy for Individuals With Chronic Pain: Efficacy, Innovations, and Directions for Research.” American Psychologist 69, no. 2: 153–166. 10.1037/a0035747. 24547801

[jan16716-bib-0018] Eriksen, M. , and T. Frandsen . 2018. “The Impact of Patient, Intervention, Comparison, Outcome (PICO) as a Search Strategy Tool on Literature Search Quality: A Systematic Review.” Journal of the Medical Library Association 106, no. 4: 420–431. 10.5195/jmla.2018.345. 30271283 PMC6148624

[jan16716-bib-0019] Gannon, J. , J. Atkinson , T. Chircop‐Rollick , et al. 2019. “Telehealth Therapy Effects of Nurses and Mental Health Professionals From 2 Randomized Controlled Trials for Chronic Back Pain.” Clinical Journal of Pain 35, no. 4: 295–303. 10.1097/ajp.0000000000000678. 30664550

[jan16716-bib-0020] Gatchel, R. J. , Y. B. Peng , M. L. Peters , P. N. Fuchs , and D. C. Turk . 2007. “The Biopsychosocial Approach to Chronic Pain: Scientific Advances and Future Directions.” Psychological Bulletin 133, no. 4: 581–624. 17592957 10.1037/0033-2909.133.4.581

[jan16716-bib-0021] Gauthier, K. , C. Dulong , and C. Argáez . 2019. Multidisciplinary Treatment Programs for Patients With Chronic Non‐Malignant Pain: A Review of Clinical Effectiveness, Cost‐Effectiveness, and Guidelines–an Update. CADTH Rapid Response Report: Summary With Critical Appraisal. Ottawa, ON: Canadian Agency for Drugs and Technologies in Health. 31449369

[jan16716-bib-0022] Geraghty, A. W. , E. Maund , D. Newell , et al. 2021. “Self‐Management for Chronic Widespread Pain Including Fibromyalgia: A Systematic Review and Meta‐Analysis.” PLoS One 16, no. 7: e0254642. 34270606 10.1371/journal.pone.0254642 PMC8284796

[jan16716-bib-0023] Gialanella, B. , L. Comini , A. Olivares , E. Gelmini , E. Ubertini , and G. Grioni . 2020. “Pain, Disability and Adherence to Home Exercises in Patients With Chronic Neck Pain: Long Term Effects of Phone Surveillance. A Randomized Controlled Study.” European Journal of Physical and Rehabilitation Medicine 56, no. 1: 5686. 10.23736/s1973-9087.19.05686-7. 31165606

[jan16716-bib-0024] Gialanella, B. , T. Ettori , S. Faustini , et al. 2017. “Home‐Based Telemedicine in Patients With Chronic Neck Pain.” American Journal of Physical Medicine & Rehabilitation 96, no. 5: 327–332. 10.1097/phm.0000000000000610. 27584139

[jan16716-bib-0027] Guliani, H. , J. Witt , V. Peynenburg , et al. 2022. “Cost‐Effectiveness of Varying Degrees and Models of Therapist‐Assisted Transdiagnostic Internet‐Delivered Cognitive Behaviour Therapy: Evidence From a Randomized Controlled Trial.” Internet Interventions 29: 100567. 10.1016/j.invent.2022.100567. 36060196 PMC9428814

[jan16716-bib-0028] Guzmán, J. , R. Esmail , K. Karjalainen , A. Malmivaara , E. Irvin , and C. Bombardier . 2001. “Multidisciplinary Rehabilitation for Chronic Low Back Pain: Systematic Review.” British Medical Journal 322, no. 7301: 1511–1516. 11420271 10.1136/bmj.322.7301.1511 PMC33389

[jan16716-bib-0029] Healthify He Puna Waiora . 2024. “Chronic Pain.” https://healthify.nz/health‐a‐z/c/chronic‐pain/.

[jan16716-bib-0030] Hestmann, R. , O. Bratås , and K. Grønning . 2023. “Chronic Pain Self‐Management Interventions in Primary Care – Does It Make any Difference? A Qualitative Study.” BMC Health Services Research 23, no. 1: 537. 10.1186/s12913-023-09548-8. 37226178 PMC10210462

[jan16716-bib-0032] Hong, Q. , S. Fàbregues , G. Bartlett , et al. 2018. “The Mixed Methods Appraisal Tool (MMAT) Version 2018 for Information Professionals and Researchers.” Education for Information 34, no. 4: 285–291. 10.3233/efi-180221.

[jan16716-bib-0033] Hussain, A. , H. Haroon , A. Ahmed , and S. A. Gilani . 2022. “Digital Technologies in Management of Chronic Pain‐ A Systematic Review.” Journal of the Pakistan Medical Association 72: 1158–1165. 35751328 10.47391/JPMA.3885

[jan16716-bib-0034] Huter, K. , T. Krick , D. Domhoff , K. Seibert , K. Wolf‐Ostermann , and H. Rothgang . 2020. “Effectiveness of Digital Technologies to Support Nursing Care: Results of a Scoping Review.” Journal of Multidisciplinary Healthcare 13: 1905–1926. 10.2147/jmdh.s286193. 33328736 PMC7734078

[jan16716-bib-0035] International Association for the study of Pain (IASP) . 2021. “Terminology.” https://www.iasp‐pain.org/resources/terminology/.

[jan16716-bib-0036] Kerns, R. , D. Burgess , B. Coleman , et al. 2022. “Self‐Management of Chronic Pain: Psychologically Guided Core Competencies for Providers.” Pain Medicine 23, no. 11: 1815–1819. 10.1093/pm/pnac083. 35642906 PMC9629397

[jan16716-bib-0037] Kostis, J. , and J. Dobrzynski . 2020. “Limitations of Randomized Clinical Trials.” American Journal of Cardiology 129: 109–115. 10.1016/j.amjcard.2020.05.011. 32560898

[jan16716-bib-0038] Kroenke, K. , F. Baye , S. G. Lourens , et al. 2019. “Automated Self‐Management (ASM) vs. ASM ‐Enhanced Collaborative Care for Chronic Pain and Mood Symptoms: The CAMMPS Randomized Clinical Trial.” Journal of General Internal Medicine 34, no. 9: 1806–1814. 10.1007/s11606-019-05121-4. 31228055 PMC6712242

[jan16716-bib-0040] Lim, J.‐A. , S.‐H. Choi , W. Lee , et al. 2018. “Cognitive‐Behavioral Therapy for Patients With Chronic Pain.” Medicine 97, no. 23: e10867. 10.1097/md.0000000000010867. 29879022 PMC5999451

[jan16716-bib-0041] Lin, J. , S. Paganini , L. Sander , et al. 2017. “An Internet‐Based Intervention for Chronic Pain.” Deutsches Ärzteblatt International 114: 681–688. 10.3238/arztebl.2017.0681. 29082858 PMC5672594

[jan16716-bib-0042] Mahoney, A. , A. Elders , I. Li , et al. 2021. “A Tale of Two Countries: Increased Uptake of Digital Mental Health Services During the COVID‐19 Pandemic in Australia and New Zealand.” Internet Interventions 25: 100439. 10.1016/j.invent.2021.100439. 34401395 PMC8350613

[jan16716-bib-0044] Martorella, G. , M. Boitor , M. Berube , S. Fredericks , S. Le May , and C. Gélinas . 2017. “Tailored Web‐Based Interventions for Pain: Systematic Review and Meta‐Analysis.” Journal of Medical Internet Research 19, no. 11: e385. 10.2196/jmir.8826. 29127076 PMC5701966

[jan16716-bib-0045] Mayhew, M. , B. Balderson , A. Cook , et al. 2023. “Comparing the Clinical and Cost ‐Effectiveness of Remote (Telehealth and Online) Cognitive Behavioral Therapy‐Based Treatments for High‐Impact Chronic Pain Relative to Usual Care: Study Protocol for the Resolve Multisite Randomized Control Trial.” Trials 24, no. 1: 196. 10.1186/s13063-023-07165-8. 36927459 PMC10018633

[jan16716-bib-0047] Milosevic, I. , D. Cameron , M. Milanovic , R. McCabe , and K. Rowa . 2021. “Face‐To‐Face Versus Video Teleconference Group Cognitive Behavioural Therapy for Anxiety and Related Disorders: A Preliminary Comparison.” Canadian Journal of Psychiatry 67, no. 5: 391–402. 10.1177/07067437211027319. 34159838 PMC9065489

[jan16716-bib-0048] Morcillo‐Muñoz, Y. , A. Sánchez‐Guarnido , S. Calzón‐Fernández , and I. amp; Baena‐Parejo . 2022. “Multimodal Chronic Pain Therapy for Adults via Smartphone: Randomized Controlled Clinical Trial.” Journal of Medical Internet Research 24, no. 5: e36114. 10.2196/36114. 35373776 PMC9133987

[jan16716-bib-0049] Morgan, C. , E. Mason , J. Newby , et al. 2017. “The Effectiveness of Unguided Internet Cognitive Behavioural Therapy for Mixed Anxiety and Depression.” Internet Interventions 10: 47–53. 10.1016/j.invent.2017.10.003. 30135752 PMC6084910

[jan16716-bib-0050] Nahin, R. L. , T. Feinberg , F. P. Kapos , and G. W. Terman . 2023. “Estimated Rates of Incident and Persistent Chronic Pain Among US Adults, 2019‐2020.” JAMA Network Open 6, no. 5: e2313563. 37191961 10.1001/jamanetworkopen.2023.13563 PMC10189566

[jan16716-bib-0051] National Institute for Health and Care Excellence . 2021. “Chronic Pain (Primary and Secondary) in Over 16s: Assessment of all Chronic Pain and Management of Chronic Primary Pain (NICE Guideline NG193).” https://www.nice.org.uk/guidance/ng193. 33939353

[jan16716-bib-0052] New Zealand Pain Society . 2024. “Navigating Pain.” https://www.nzps.org.nz/painresource/.

[jan16716-bib-0053] Newby, J. , E. Mason , N. Kladnistki , et al. 2021. “Integrating Internet CBT Into Clinical Practice: A Practical Guide for Clinicians.” Clinical Psychologist 25, no. 2: 164–178. 10.1080/13284207.2020.1843968.

[jan16716-bib-0054] Ouzzani, M. , H. Hammady , Z. Fedorowicz , and A. Elmagarmid . 2016. “Rayyan—A Web and Mobile App for Systematic Reviews.” Systematic Reviews 5, no. 1: 384. 10.1186/s13643-016-0384-4. PMC5139140 27919275

[jan16716-bib-0055] Page, M. , J. McKenzie , P. Bossuyt , et al. 2021. “The Prisma 2020 Statement: An Updated Guideline for Reporting Systematic Reviews.” BMJ 372: 71. 10.1136/bmj.n71. PMC8005924 33782057

[jan16716-bib-0057] Perez, J. , K. Niburski , M. Stoopler , and P. Ingelmo . 2021. “Telehealth and Chronic Pain Management From Rapid Adaptation to Long‐Term Implementation in Pain Medicine: A Narrative Review.” PAIN Reports 6, no. 1: e912. 10.1097/pr9.0000000000000912. 33981934 PMC8108593

[jan16716-bib-0058] Rejula, V. , J. Anitha , R. Belfin , and J. Peter . 2021. “Chronic Pain Treatment and Digital Health Era‐An Opinion.” Frontiers in Public Health 9, no. 10: 3389. 10.3389/fpubh.2021.779328 PMC8702955 34957031

[jan16716-bib-0059] Rutledge, T. , J. H. Atkinson , R. Holloway , et al. 2018. “Randomized Controlled Trial of Nurse‐Delivered Cognitive‐Behavioral Therapy Versus Supportive Psychotherapy Telehealth Interventions for Chronic Back Pain.” Journal of Pain 19, no. 9: 1033–1039. 10.1016/j.jpain.2018.03.017. 29673974

[jan16716-bib-0060] Scottish Intercollegiate Guidelines Network . 2019. “Management of Chronic Pain (SIGN 136).” https://www.sign.ac.uk/media/2097/sign136_2019.pdf.

[jan16716-bib-0061] Seibert, K. , D. Domhoff , K. Huter , T. Krick , H. Rothgang , and K. Wolf‐Ostermann . 2020. “Application of Digital Technologies in Nursing Practice: Results of a Mixed Methods Study on nurses' Experiences, Needs and Perspectives.” Zeitschrift für Evidenz, Fortbildung und Qualität im Gesundheitswesen 158–159: 94–106. 10.1016/j.zefq.2020.10.010. 33223491

[jan16716-bib-0069] Skolasky, R. L. , S. Nolan , R. Pierre , P. Vinch , and J. L. Taylor . 2024. “Nurse‐Led Web‐Based Self‐Management Program to Improve Patient Activation and Health Outcomes in Patients With Chronic Low Back Pain: An Acceptability and Feasibility Pilot Study.” BMC Nursing 23, no. 1: 524. 39085831 10.1186/s12912-024-02155-w PMC11293200

[jan16716-bib-0064] Staudt, M. D. 2022. “The Multidisciplinary Team in Pain Management.” Neurosurgery Clinics of North America 33, no. 3: 241–249. 10.1016/j.nec.2022.02.002. 35718393

[jan16716-bib-0065] Treede, R. D. , W. Rief , A. Barke , et al. 2015. “A Classification of Chronic Pain for ICD‐11.” Pain 156, no. 6: 1003–1007. 10.1097/j.pain.0000000000000160. 25844555 PMC4450869

[jan16716-bib-0066] Turk, D. C. , H. D. Wilson , and A. Cahana . 2011. “Treatment of Chronic Non‐Cancer Pain.” Lancet 377, no. 9784: 2226–2235. 10.1016/S0140-6736(11)60402-9. 21704872

[jan16716-bib-0067] Whittemore, R. , and K. Knafl . 2005. “The Integrative Review: Updated Methodology.” Journal of Advanced Nursing 52, no. 5: 546–553. 10.1111/j.1365-2648.2005.03621.x. 16268861

[jan16716-bib-0068] Zilcha‐Mano, S. , S. Roose , P. Brown , and B. Rutherford . 2019. “Not Just Nonspecific Factors: The Roles of Alliance and Expectancy in Treatment, and Their Neurobiological Underpinnings.” Frontiers in Behavioral Neuroscience 12: 293. 10.3389/fnbeh.2018.00293. 30760986 PMC6361734

